# Comparison of Diagnostic Accuracy of Extended Focused Assessment of Sonography in Trauma (eFAST) With Clinical Examination and Chest X-ray in Detecting Hemo/Pneumothorax for Blunt Chest Trauma

**DOI:** 10.7759/cureus.96908

**Published:** 2025-11-15

**Authors:** Mohammed Adam S, Sandeep Samson, Nirmal Kumar, Srihari C, Minaa Alrawe

**Affiliations:** 1 General Surgery, Pondicherry Institute of Medical Sciences, Kalapet, IND; 2 General Surgery, James Cook University Hospital, Middlesbrough, GBR

**Keywords:** blunt chest trauma, diagnostic accuracy, extended focused assessment with sonography in trauma, hemothorax, pneumothorax

## Abstract

Background

Blunt chest trauma represents a significant cause of preventable mortality globally, accounting for approximately one-third of trauma-related deaths in India. Timely diagnosis of hemothorax and pneumothorax is essential to prevent clinical deterioration. While clinical examination and chest X-ray (CXR) are commonly employed, their diagnostic sensitivity is limited. The extended focused assessment with sonography in trauma (eFAST) offers a rapid, bedside, radiation-free alternative with demonstrated diagnostic utility.

Objective

The objective of this study was to evaluate and compare the diagnostic accuracy of eFAST, clinical examination, and CXR for the detection of hemothorax and pneumothorax, with CT chest serving as the reference standard.

Methods

This prospective diagnostic observational study was conducted at the Department of General Surgery, Pondicherry Institute of Medical Sciences, India, between January 2021 and June 2022. Eighty-one hemodynamically stable patients with blunt chest trauma underwent clinical examination, CXR, eFAST, and CT chest evaluation. Diagnostic performance metrics, sensitivity, specificity, positive predictive value (PPV), negative predictive value (NPV), and accuracy, were calculated with CT chest as the gold standard.

Results

The mean age was 49.2 ± 15.6 years, with 71.6% of participants being male. Road traffic accidents were the predominant mechanism (67.9%). CT chest revealed thoracic injuries in 64.2% of cases. Clinical examination showed a sensitivity of 34.6% and specificity of 100%. Chest X-ray had a sensitivity of 76.9%, a specificity of 85.2%, and an accuracy of 81.5%. eFAST achieved a sensitivity of 98.1%, specificity of 98.8%, PPV of 98%, NPV of 96.7%, and an overall accuracy of 98.5%.

Conclusion

eFAST demonstrated superior diagnostic accuracy compared to clinical examination and CXR, and closely approximated the diagnostic reliability of CT chest. Given its portability, absence of radiation, and rapid application, eFAST should be integrated as a frontline tool in the evaluation of blunt chest trauma.

## Introduction

Trauma accounts for approximately 8% of global mortality and remains a major cause of death among younger populations. Among the five leading causes of death in individuals under 30 years, three, including road traffic accidents, homicide, and suicide, are trauma-related [1,2]. The burden is especially pronounced in low- and middle-income countries [3]. Hemorrhage contributes to nearly 30-40% of trauma-related deaths [4,5], with thoracic injuries responsible for nearly one-third of trauma fatalities in India [6]. Blunt chest trauma can result in significant intrathoracic or intra-abdominal hemorrhage. Up to 12% of these cases involve intra-peritoneal bleeding [7]. Pneumothorax, although potentially preventable, remains a significant contributor to trauma-related mortality [8]. The rising incidence of high-velocity traffic accidents, poor enforcement of safety regulations, and increased urban crowding have further contributed to the burden of thoracic trauma in India. Delay in diagnosis of internal hemorrhage increases the risk of death by approximately 1% every three minutes [9,10]. Although diagnostic peritoneal lavage (DPL) was historically used for detecting intra-abdominal bleeding, it has been largely replaced due to its invasive nature and associated risks [11]. Chest X-Ray (CXR) remains a commonly used first-line imaging modality in trauma evaluation. However, its diagnostic sensitivity is limited, especially in supine positioning and in detecting small pneumothoraces [12]. Point-of-care ultrasonography (POCUS) has emerged as a valuable bedside screening tool. The extended focused assessment with sonography in trauma (eFAST) expands upon traditional FAST by incorporating thoracic views, facilitating detection of both hemothorax and pneumothorax [13]. eFAST is portable, non-invasive, and avoids radiation exposure. Prior studies have demonstrated its higher sensitivity compared to CXR while maintaining high specificity [6,14]. Though operator experience influences results, studies suggest that thoracic ultrasound has a relatively short learning curve [15,16].

Currently, CT chest is considered the gold standard for detecting hemothorax and pneumothorax [17], yet its use in emergency settings is often limited by availability, cost, patient instability, and radiation exposure. Despite multiple international studies validating the utility of eFAST, there remains limited evidence from Indian trauma settings directly comparing its diagnostic performance with that of CXR and clinical examination using CT as the reference standard. Recognizing these gaps, the present study was undertaken to evaluate the diagnostic accuracy of eFAST in detecting hemothorax and pneumothorax in patients with blunt chest trauma, while comparing its performance against CXR and clinical examination, thereby addressing the need for a rapid, reliable, and noninvasive diagnostic tool in acute trauma care.

## Materials and methods

Study design and setting

This was a prospective diagnostic observational study conducted in the Department of General Surgery at Pondicherry Institute of Medical Sciences (PIMS), Kalapet, Pondicherry, India, a 740-bed tertiary-care teaching hospital in South India. The study duration extended from January 2021 to June 2022.

Participants

All patients presenting to the emergency department with blunt chest trauma were screened. Only hemodynamically stable patients were included. Inclusion criteria included patients aged ≥18 years who were hemodynamically stable with a history of blunt chest trauma. Hemodynamically unstable patients requiring immediate intervention, patients with previous chest tube insertion, penetrating injuries to the chest, and pregnant women were excluded.

Outcome measures

The primary outcome was the diagnostic accuracy of eFAST in detecting hemothorax and pneumothorax, using CT chest as the reference standard. The secondary outcome was the diagnostic accuracy of clinical examination and CXR against CT chest in detecting thoracic injuries.

CT chest protocol

Contrast-enhanced CT chest was performed only in patients with clinical indications for thoracic injury, such as suspected pneumothorax, hemothorax, lung contusion, or rib fractures. All scans were performed as part of routine institutional trauma care, not specifically for the study. Patients were positioned supine, and images were acquired with 5-mm axial slices. CT served as the reference standard for comparison.

eFAST scanning protocol

eFAST examinations were performed at the bedside using a 3.5-5 MHz convex transducer in the supine position. The scanning protocol included (i) Thoracic views: anterior chest (mid-clavicular line, 2nd-4th intercostal spaces), lateral chest (mid-axillary line, 5th-8th intercostal spaces), and diaphragmatic windows to assess for pneumothorax or hemothorax, and (ii) Abdominal views: hepatorenal, splenorenal, pelvic, and subxiphoid pericardial windows as per standard FAST protocol.

All scans were performed by radiology residents trained in trauma ultrasonography, as they are the first-line operators for trauma ultrasonography in our institution, ensuring consistency and image quality under senior supervision.

Blinding and diagnostic workflow

eFAST was performed immediately upon patient arrival, followed by clinical examination and CXR. CT chest was performed within 30-60 minutes of the eFAST study when clinically indicated, and all imaging was completed within the first two hours of emergency presentation. Radiologists interpreting CT scans were blinded to eFAST and CXR results, ensuring diagnostic independence.

Sample size

A minimum required sample size of 81 patients was calculated using prior data from Devadoss et al. [6], assuming an expected sensitivity of eFAST at 88.6%, CT chest at 99.1%, with 80% statistical power and a 5% significance level. The calculation was based on the comparison of two correlated proportions using McNemar's test for paired proportions. A consecutive sampling method was adopted to minimize selection bias. All patients meeting the inclusion and exclusion criteria during the study period were enrolled.

Study procedure

Following informed consent, demographic information and the mechanism of injury were recorded for each patient. A standardized sequence of assessments was followed as in Table 1.

**Table 1:** Sequence of assessments eFAST: extended focused assessment with sonography in trauma; CXR: chest X-ray; AP: anteroposterior

Step	Modality	Parameters Observed
1	Clinical Examination	Chest tenderness, decreased air entry (bilaterally)
2	Chest X-ray (CXR)	Supine AP view: costophrenic angle blunting, rib fractures, opacities
3	eFAST	Conducted with portable ultrasound (5–10 MHz linear/convex probes). Pneumothorax diagnosed by absent lung sliding/barcode sign; hemothorax by anechoic pleural fluid
4	CT Chest (Gold Standard)	Multidetector contrast-enhanced CT interpreted by radiologists blinded to eFAST/CXR findings

Data collection and handling

Demographic data, mechanism of injury, clinical findings, eFAST and CT results, and patient outcomes were recorded using a pre-tested proforma. No missing data were encountered, as all included patients had complete eFAST, CXR, and CT findings.

Statistical analysis

Data were analyzed using IBM SPSS Statistics for Windows, version 23 (IBM Corp., Armonk, New York, United States). Continuous variables were expressed as mean ± standard deviation (SD) or median [IQR], and categorical variables as frequencies (%). Normality of continuous variables was assessed using the Shapiro-Wilk test. Comparisons between diagnostic modalities were performed using McNemar’s test for paired proportions. Diagnostic indices, including sensitivity, specificity, positive predictive value (PPV), negative predictive value (NPV), and accuracy, were calculated with 95% confidence intervals (CI). A p-value < 0.05 was considered statistically significant.

Ethical considerations

This study was approved by the Institute Ethics Committee of PIMS (approval number: IEC: RC/2022/71). Written informed consent was obtained from all participants or their legal guardians.

## Results

Demographic characteristics

As shown in Table 2, a total of 81 hemodynamically stable patients with blunt chest trauma were enrolled. The mean age of participants was 49.2 ± 15.6 years (range: 14-83 years). The majority were male (71.6%, n = 58), and females accounted for 28.4% (n = 23).

**Table 2:** Distribution of participants according to sex (N=81)

Sex	Frequency	Percentage
Male	58	71.60%
Female	23	28.40%

Mechanism of injury

The most common cause of injury was RTA, accounting for 67.9% of cases (n = 55), followed by self-falls (22.2%, n = 18), assault (6.2%, n = 5), and other causes, including bull hits and falls from height (3.7%, n = 3) (Table 3).

**Table 3:** Mechanisms of injury

Mechanism	Frequency	Percentage
Road Traffic Accident	55	67.90%
Self-Fall	18	22.20%
Assault	5	6.20%
Other (e.g., bull hit, fall from height)	3	3.70%

Clinical findings

All patients (100%) presented with chest tenderness. However, only 22.2% (n = 18) had decreased air entry on auscultation. Notably, all patients with decreased air entry had corresponding abnormalities detected by eFAST.

CXR findings

CXR findings were categorized based on the presence of costophrenic (CP) angle blunting and rib fractures, as shown in Table 4. 

**Table 4:** Chest X-ray findings CP: costophrenic

CXR Findings	Frequency	Percentage
Normal/Clear	31	38.20%
Only CP Angle Blunting	7	8.60%
Only Rib Fracture	10	12.30%
Both CP Blunting + Rib Fracture	33	40.70%

Overall, CP-angle blunting (only CP blunting and CP blunting + rib fracture) was observed in 49.4% (n = 40), and rib fractures (Only rib fracture and CP blunting + rib fracture) were seen in 53.1% (n = 43).

eFAST findings

**Figure 1:**
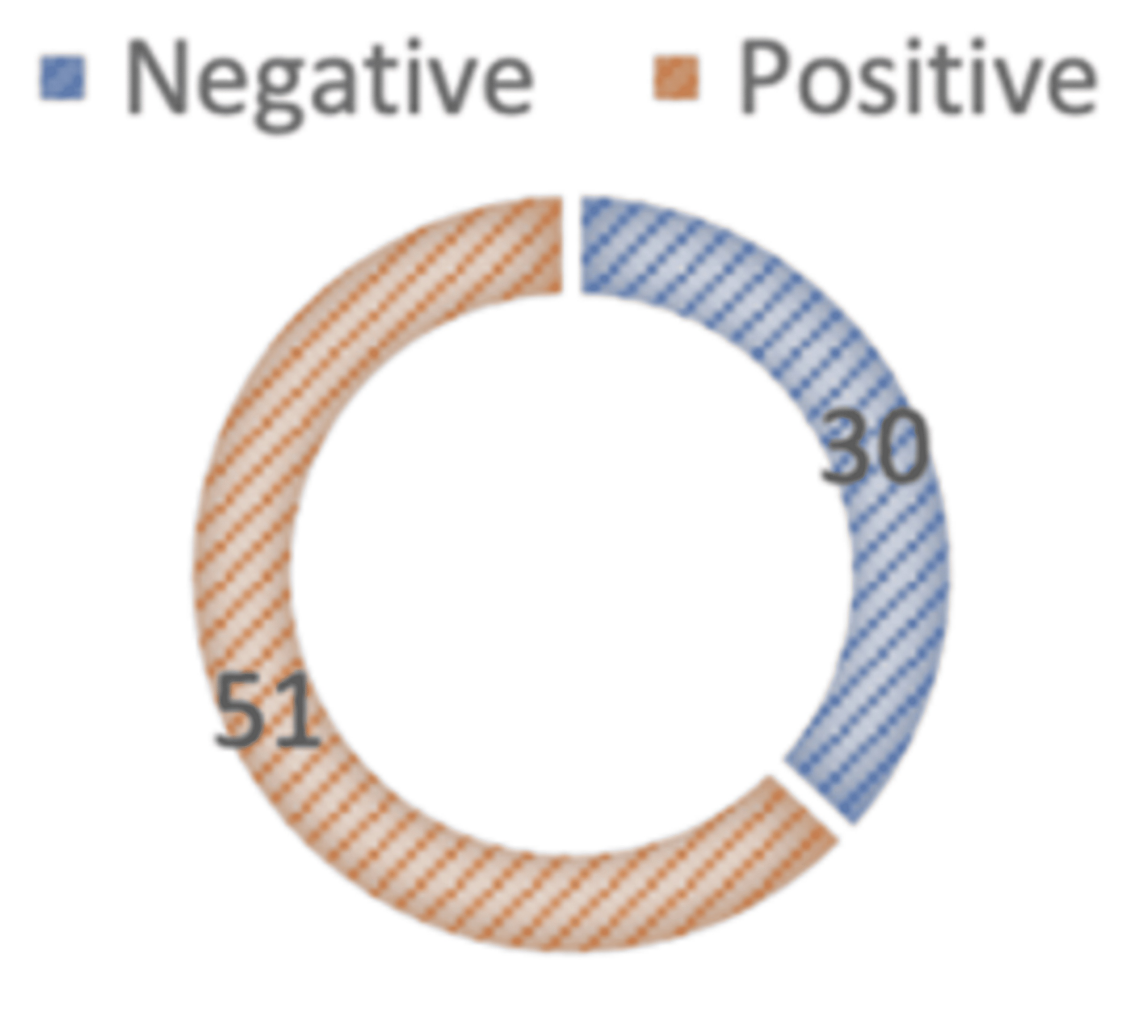
Distribution of participants according to anechoic pleural fluid (haemothorax) being detected in eFAST eFAST: extended focused assessment with sonography in trauma Data is shown in percentage

eFAST identified abnormalities in 63% of patients (n = 51). Common sonographic signs included anechoic pleural fluid indicating hemothorax and absent lung sliding with a "barcode sign" ("Stratosphere sign") indicative of pneumothorax. eFAST remained positive even in several patients with normal CXR findings, as depicted in Figure 1.

CT chest findings (gold standard)

CT chest identified thoracic abnormalities in 64.2% of cases (n = 52), while 35.8% (n = 29) had normal findings. Specific findings included as shown in Table 5.

**Table 5:** CT chest findings Note: Several patients had combined hemothorax and pneumothorax.

Finding	Frequency	Percentage
Normal	29	35.80%
Hemothorax	52	64.20%
Pneumothorax	20	24.70%
Bilateral Involvement	16	30.8% (of abnormal cases)

The extent of hemothorax and pneumothorax was assessed qualitatively on eFAST and confirmed by CT. The majority of hemothoraces were small to moderate in size, occupying less than one-third of the pleural cavity, while pneumothoraces were minimal to moderate, involving less than 20% of the hemithorax.

Interventions and outcomes

Intercostal drainage (ICD) was performed in 24.7% (n = 20). The remaining 75.3% (n = 61) were managed conservatively. A total of 52 participants (64.2%) had favorable outcomes and were discharged, and 35.8% (n = 29) were discharged against medical advice (DAMA) (Table 6).

**Table 6:** Interventions and outcomes ICD: intercostal drainage

Parameter	Frequency	Percentage
ICD Insertion	20	24.70%
Conservative Management	61	75.30%
Recovered/Discharged	52	64.20%
Discharged Against Medical Advice	29	35.80%

Comparison between diagnostic modalities

**Figure 2:**
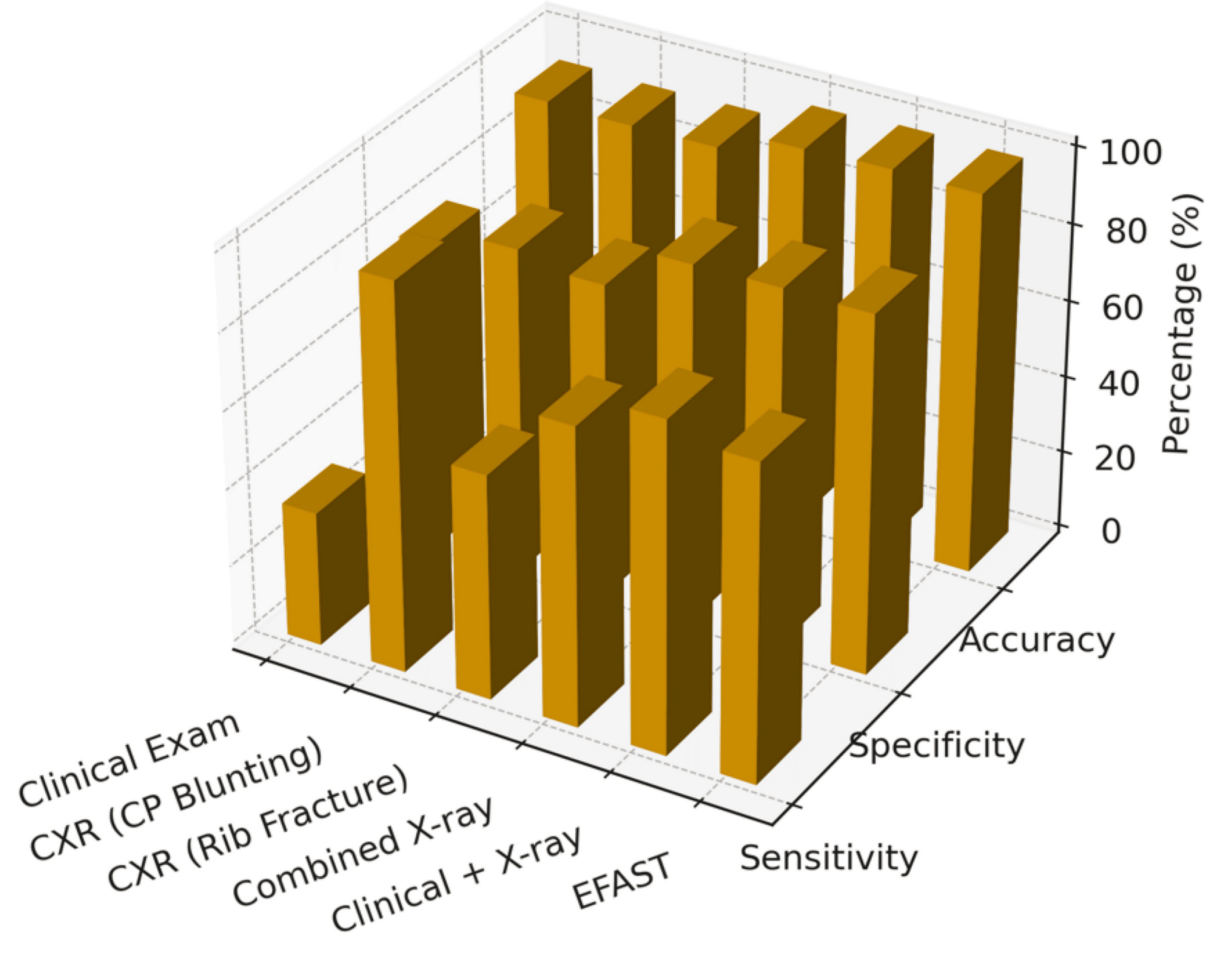
Comparison of diagnostic accuracy between modalities CXR: chest X-ray; EFAST: extended focused assessment with sonography in trauma; CP: costophrenic

As shown in Table 7 and Figure 2, eFAST demonstrated the highest diagnostic performance among all modalities. Compared with CT chest, it achieved a sensitivity of 98.1% (95%CI: 89.9-99.9), specificity of 98.8% (95%CI: 92.4-100), PPV of 98.0% (95%CI: 89.4-99.9), NPV of 96.7% (95%CI: 88.7-99.6), and overall accuracy of 98.5% (95%CI: 92.0-99.9). eFAST missed one case of a small posterior pleural collection (minimal hemothorax) that was identified on CT. The lesion was clinically insignificant and located in a region less accessible to ultrasound in supine trauma patients. CXR showed moderate diagnostic capability with a sensitivity of 76.9% (95%CI: 64.3-87.1) and specificity of 85.2% (95%CI: 73.0-93.4). Clinical examination performed poorly with a sensitivity of 34.6% (95%CI: 23.1-47.8) and specificity of 100% (95%CI: 92.0-100). McNemar’s test indicated a statistically significant difference between eFAST and CXR (p < 0.001), confirming eFAST’s superior diagnostic accuracy. The difference between eFAST and clinical examination was also highly significant (p < 0.001), whereas CXR showed a modest but significant improvement over clinical findings (p = 0.004).

**Table 7:** Comparison of diagnostic accuracy between modalities *p-value < 0.05 was considered statistically significant eFAST: extended focused assessment with sonography in trauma; CP: costophrenic; PPV: positive predictive value; NPV: negative predictive value

Modality	Sensitivity (%) (95% CI)	Specificity (%) (95% CI)	PPV (%) (95% CI)	NPV (%) (95% CI)	Accuracy (%) (95% CI)	McNemar’s test (p-value)
Clinical Examination	34.6 (23.1–47.8)	100 (92.0–100)	100 (78.1–100)	55.3 (44.2–66.1)	58.0 (46.8–68.6)	—
Chest X-Ray (CP Angle Blunting)	76.9 (64.3–87.1)	85.2 (73.0–93.4)	87.8 (76.7–94.4)	72.0 (58.8–82.5)	81.5 (71.2–89.2)	0.004*
Combined X-Ray Findings	92.3 (82.4–97.7)	92.6 (83.6–97.9)	96.0 (86.5–99.5)	87.1 (75.2–94.3)	92.4 (84.2–97.2)	0.001*
eFAST	98.1 (89.9–99.9)	98.8 (92.4–100)	98.0 (89.4–99.9)	96.7 (88.7–99.6)	98.5 (92.0–99.9)	<0.001*

## Discussion

This prospective study evaluated and compared the diagnostic accuracy of eFAST, clinical examination, and CXR in detecting hemothorax and pneumothorax in hemodynamically stable patients presenting with blunt chest trauma. The CT chest was used as the gold standard for comparison.

eFAST demonstrated the highest diagnostic performance among the modalities evaluated, achieving a sensitivity of 98.1%, a specificity of 98.8%, and an accuracy of 98.5%. These findings align with those reported by Devadoss et al. in a similar Indian cohort, where eFAST showed high sensitivity and specificity for chest trauma detection [6]. Similarly, Basnet et al. observed comparable results in a large trauma population, further validating eFAST's diagnostic utility [18]. In contrast, clinical examination alone showed low sensitivity (34.6%) but perfect specificity (100%). This reflects earlier concerns raised by Abdalla et al., who emphasized that clinical signs, especially in early or minor thoracic injuries, are unreliable as sole diagnostic indicators [19].

CXR, a commonly used initial imaging tool in trauma evaluation, demonstrated moderate sensitivity (76.9%) and specificity (85.2%) when evaluated individually for CP-angle blunting or rib fractures. However, when both features were considered in combination, diagnostic accuracy improved to 92.4%. Despite this improvement, eFAST remained superior, particularly in detecting injuries missed by X-ray, such as small pneumothoraces or bilateral involvement. These findings are supported by prior research. A systematic review by Stengel et al. concluded that point-of-care ultrasound, especially eFAST, has high diagnostic accuracy for thoracoabdominal trauma, outperforming traditional imaging in many scenarios [20]. Kirkpatrick et al. demonstrated that thoracic eFAST surpassed supine CXR in sensitivity for post-traumatic pneumothorax, while maintaining excellent specificity [21].

Importantly, Sauter et al. highlighted that the few cases missed by eFAST were typically minimal pneumothoraces with no need for urgent intervention [22], suggesting that in most clinical contexts, missing such findings may not alter immediate management. This supports the practical utility of eFAST even in settings where CT is not immediately available. In another Indian study by Bhoil et al., eFAST demonstrated higher sensitivity than supine CXRs in diagnosing traumatic pneumothorax, aligning with the present study’s findings [23].

Given this growing body of evidence, eFAST can be confidently considered a frontline diagnostic modality in the early evaluation of blunt chest trauma, particularly in emergency departments and low-resource settings. It offers advantages such as portability, lack of radiation exposure, repeatability, and real-time bedside application.

Strengths and limitations

Strengths of this study include its prospective design, use of a standardized diagnostic sequence, and comparison of all modalities against a blinded CT chest as the reference standard. Both hemothorax and pneumothorax were evaluated separately and combined, providing a comprehensive diagnostic picture.

However, several limitations must be acknowledged. The study was single-center with a relatively modest sample size (n=81), potentially limiting generalizability. Also, only hemodynamically stable patients were included, excluding those in critical condition who might benefit most from rapid bedside imaging. Operator dependency is inherent to eFAST. Although performed by trained radiology residents under supervision, variability in skill may have influenced results. Inter-observer variability was not formally analyzed, and findings may not fully account for learning curves or subtle diagnostic differences. The 35.8% of patients who got discharged against medical advice introduces potential outcome bias, as follow-up imaging or interventions may have revealed complications or missed injuries. Additionally, the 30-60 minute interval between eFAST and CT could introduce minor bias due to evolving physiological changes.

Clinical implications

This study confirms the utility of eFAST as a highly accurate, safe, and rapid diagnostic tool for detecting life-threatening thoracic injuries in blunt trauma. It has the potential to reduce reliance on radiographic imaging, shorten time to diagnosis and intervention, improve patient triage and prioritization, and serve as a cost-effective solution in resource-limited settings. Given its advantages, eFAST training should be emphasized in trauma and emergency medicine curricula. Standardized operator training, periodic re-evaluation, and inclusion of inter-observer reliability assessments could further enhance eFAST's reliability.

## Conclusions

In the evaluation of blunt chest trauma, eFAST proved to be a highly accurate, rapid, and non-invasive diagnostic tool for detecting hemothorax and pneumothorax. When compared with clinical examination and CXR, eFAST demonstrated superior diagnostic performance, achieving a sensitivity of 98.1% and a specificity of 98.8%, closely approximating the diagnostic reliability of CT chest, the gold standard in this study. It effectively detected bilateral and subtle pathologies that were missed by clinical and radiographic evaluation, reinforcing its role as a reliable point-of-care imaging technique in emergency settings.

eFAST’s portability, repeatability, and absence of radiation make it a reliable tool for first-line use in resource-limited or high-volume trauma centers. Implementation of structured eFAST training programs for emergency and surgical personnel, along with periodic competency evaluation, can further enhance diagnostic accuracy and optimize trauma management outcomes. Future multicentric studies incorporating larger cohorts, including both stable and unstable trauma patients, are warranted to validate these findings and strengthen evidence-based trauma protocols.
